# The relationship between perceived service quality and patient willingness to recommend at a national oncology hospital network

**DOI:** 10.1186/1472-6963-11-46

**Published:** 2011-02-25

**Authors:** Christopher G Lis, Mark Rodeghier, Digant Gupta

**Affiliations:** 1 Cancer Treatment Centers of America® (CTCA), 500 E. Remington St., Schaumburg, IL 60173, USA

## Abstract

**Background:**

"Willingness to recommend" questions are being increasingly used to measure and manage patient loyalty. Yet, there is little data in the literature correlating the "willingness to recommend" question with commonly used perceived service quality items in surveys to identify the key drivers of the optimal patient experience. We therefore evaluated the relationship between perceived service quality and subsequent single top box "willingness to recommend" scores among oncology patients.

**Methods:**

A total of 2018 returning cancer patients treated at Cancer Treatment Centers of America^® ^(CTCA) responded to an internally developed service quality questionnaire, which covered the following dimensions: operations and services, treatment and care with a multidisciplinary team and patient endorsements. Items were measured on a 7-point Likert-type scale ranging from "completely dissatisfied" to "completely satisfied." Patient willingness to, "recommend this facility to friends and associates" was measured on an 11-point scale ranging from "not at all likely" to "extremely likely", which was subsequently dichotomized into two categories: top box response (10) versus all others (0-9). The relationship between perceived service quality and "willingness to recommend" was assessed via Kendall's tau b correlation and univariate and multivariate logistic regression.

**Results:**

Of the 2018 patients, 959 were newly diagnosed while 1059 were previously treated. 902 were males and 1116 females. The mean age was 54.2 years and the most frequent diagnoses were breast (412), lung (294), prostate (260), colorectal (179) and pancreas (169). 1553 patients said they were "extremely likely" to recommend CTCA to friends and associates, resulting in 77% "top box" responses while 465 (23%) responded in all other categories. The key service quality drivers that were statistically significant in the final logistic model were "team helping you understand your medical condition", "staff genuinely caring for you as an individual", "whole person approach to patient care" and "CTCA medical oncologist."

**Conclusions:**

In this multi-center study, we demonstrate the predictive significance of perceived service quality as it relates to patient willingness to recommend an oncology service provider. This study is unique in reporting on the role of perceived service quality as a predictor of patient willingness to recommend in a large sample of cancer patients.

## Background

As consumerism continues to increase in healthcare, there has been a rise in awareness about how patients perceive the quality of the services they receive at a health-care institution [1,2]. In addition, the web offers patients the opportunity to shop for the best places for care due to the rise in transparency of provider information on service quality and patient experience. As a consequence, patient satisfaction with service quality is becoming an increasingly important tool for providers to demonstrate patient focus and differentiation in the marketplace, as well as enhance patient loyalty. Furthermore, providers are using the information to make important decisions regarding operational and treatment plans [3].

Evaluations of service quality provide important data on the patient's perception of the quality of care and treatment delivered by physicians, paramedical staff and the hospital as a whole [4]. Health providers can use data on service quality to design and track quality improvement over time and compare themselves to other health providers when the same measures are used, as well as recognize and expeditiously resolve service problems in real-time [5,6]. Measuring service quality also helps health care providers identify specific, and often unmet needs of patients, which has been a large focus of our work, and demonstrated by other research [7].

Similar to other acute health-care settings, the assessment of service quality, as perceived by patients, is critical in the oncology setting as well. Advances in diagnostics, treatment, supportive care and rehabilitation all necessitate continued monitoring to determine whether patients are satisfied with the increasingly complex and multidisciplinary nature of health care services that they are receiving, and to identify areas in which improvement is needed. Similar to other health-care disciplines, evaluation of perceived service quality in an acute care oncology setting, involves a diverse array of methodologies including longitudinal surveys, in-depth interviews, focus-group discussions, patient panels, consultation of voluntary groups, and analyses of patient feedback and concerns, followed by quick improvements to operations to help patients while they are undergoing care throughout the full cycle of treatment and follow up, as well as to help future patients. Patient-reported service quality surveys still continue to be the most widely used method of objectively and systematically determining a cancer patient's perception of the healthcare received. Cancer patients should be surveyed regularly due to the often aggressive nature of the disease and treatment. The modes of therapies have their own side effects and often result in difficult patient compliance. As a result, considerable demands are placed on health care providers to satisfy the complex healthcare needs of cancer patients.

The literature shows that perceived service quality can act as a marker for patient willingness to comply with the treatment plan as well as to predict a patient's willingness to recommend a provider to friends and relatives [8,9]. This is especially important in many countries where service quality data are not readily published and recommendations from family or friends becomes an important source of information for selecting a provider [9,10]. There are several studies in the literature that have evaluated service quality in cancers like gastro esophageal [11], breast [5,12], colorectal [13], lung, prostate [14] and gynecological [15,16]. Collectively, these studies have found that satisfaction with the information provided by medical staff about a patient's illness and the course of treatment is important. This is followed closely by the time spent with the physician and the interpersonal skills of the physician. Other key factors are waiting time to get an appointment, empathy of staff with the patient, the continuity of care provided, and satisfaction with the nursing staff [17]. We are unaware of any information in the oncology literature demonstrating a link between perceived service quality and patient willingness to recommend a provider. In light of the importance of this information to the healthcare industry, as well as with the goal of taking the existing research in this area to the next level, we designed a study to investigate the relationship between perceived service quality and patient willingness to recommend at a network of national oncology hospitals.

## Methods

### Study Population

All returning treating patients were eligible for inclusion in this study. Patients with all stages of all cancer types were eligible for the study. Specifically, patients who participated in the study were randomly selected from a population that had not responded to a service quality questionnaire within 60 days of the start of the study. The selected patients were approached onsite for survey administration. The surveyed cohort included 2018 randomly-selected returning cancer patients who had been treated at one of three Cancer Treatment Centers of America^® ^(CTCA) hospitals between July 2007 and September 2009. The study was approved by the CTCA Institutional Review Board.

### Questionnaire and Survey Administration

The service quality questionnaire used in this study was first developed and implemented by the Research team at CTCA in August 2006. The questionnaire was developed based on a patient-centered approach that used questions that patients view as important in their treatment experience. In addition to patient focus groups, survey dimensions were collated from several existing studies or questionnaires of oncology patients [18-21]. This service quality questionnaire covers the following dimensions of patient satisfaction: hospital operations and services, physicians and staff, and patient endorsements for others (friends and associates). After the patient consented to complete the survey, the Survey Research Associate completed the "office use only section" on the last page of the survey which includes unique patient identifiers. The survey was then given to the patient. The Survey Research Associate then opened and explained the survey, specifically describing the rating scale and the open-ended questions. Next, the Survey Research Associate informed the patient that he/she will return to collect the survey and/or explained the option of the comment/suggestion drop box. Throughout the day, the Survey Research Associate updated the survey tracking list to note the following: patients contacted, surveys returned, surveys declined, and missed patients.

### Statistical Analysis

Patient willingness to recommend, "will you recommend this facility to friends and associates?" was used as the dependent variable and was measured on an 11-point scale ranging from "not at all likely" to "extremely likely". This question is used to calculate the Net Promoter Score [22,23], a measure that has been shown in a number of industries to effectively measure customer loyalty, with increasing use in healthcare, including our hospitals as a management tool. For the purpose of this analysis, as well as in accordance with previously reported research [9,10], data were dichotomized into 2 categories: top box response (10) versus all others (0-9). The service quality items that were used as independent variables in this study were the ease of the admission (registration) process, the speed of the admission (registration) process, the timeliness with which care was delivered, team helping you understand your medical condition, team explaining your treatment options, team involving you in decision making, the amount of time spent team with you, team calling you by your name, team genuinely caring for you as an individual, team providing you with a sense of well-being, our team's "whole person" approach to patient care and the CTCA medical oncologist (patient's primary physician). These items were measured on a 7-point Likert-type scale ranging from "completely dissatisfied" to "completely satisfied." Each service quality item was also dichotomized into 2 categories: "completely satisfied" (7) and "not completely satisfied" (1-6). Other control variables that were investigated for their relationship with patient willingness to recommend were age at diagnosis, prior treatment history and gender. The prior treatment history variable categorized patients into those who have received definitive cancer treatment elsewhere before coming to CTCA and those who were newly diagnosed at CTCA. The multivariate analysis also adjusted for the effects of CTCA center and survey year with dummy variables representing these categories.

Descriptive statistics and frequencies were computed for each service quality item in the questionnaire. The relationship between perceived service quality and "willingness to recommend" was initially assessed via Kendall's tau b correlation and univariate logistic regression. Kendall's tau b is an appropriate measure of association for categorical variables and is commonly used when both variables have the same number of categories. Logistic regression was then employed to develop a multivariate model to predict patient willingness to recommend. Potential multicollinearity was assessed in two steps. Large values (above 0.70) of tau b were used as an initial screen for pairs of service quality measures, with one member of the pair not entered into the multivariate model (the measure that was more meaningful or actionable was retained). As a second check, the variance inflation factor was used with the final model to verify that multicollinearity was not significantly influencing model coefficients.

The effect of perceived service quality on patient willingness to recommend was expressed as odds ratios (ORs) with 95% confidence intervals. A difference was considered to be statistically significant if the p value was less than or equal to 0.05. All data were analyzed using SPSS version 17.0 (SPSS, Chicago, IL, USA).

## Results

### Response Rate

A total of 2754 returning patients were contacted at all three centers combined to participate in the survey between July 2007 and September 2009. However, only 2018 patients responded. As a result, the response rate for this study was 73.3%.

### Baseline Patient Characteristics

Table 1 displays baseline patient characteristics across the entire study population (N = 2018). The most frequent diagnoses were breast (N = 412), lung (N = 294), prostate (N = 260), colorectal (N = 179) and pancreatic (N = 169) cancer.

**Table 1:** Baseline Patient Characteristics (N = 2018)

Variable	Categories	Number (Percent)
Age	Mean	54.2
	Median	54.0
	Range	16-92

CTCA Center	Midwestern	953 (47.2)
	Southwestern	620 (30.7)
	Eastern	445 (22.1)

Gender	Male	902 (44.6)
	Female	1116 (55.4)

Treatment History	Newly Diagnosed	959 (47.5)
	Previously Treated	1059 (52.5)

Survey Year	2007	561 (27.8)
	2008	708 (35.1)
	2009	749 (37.1)

### Service Quality Items

Table 2 describes the level of patient satisfaction with service quality items concerning CTCA operations and services. Table 3 describes the level of patient satisfaction with service quality items concerning CTCA's multidisciplinary patient care team. Table 4 reports the patient willingness to recommend CTCA to friends and associates. 1553 (77.0%) patients said they were "extremely likely" to recommend CTCA to friends and associates.

**Table 2:** Service Quality Items: Operations and Services

How satisfied are you with:	*Completely Satisfied*	*Not Completely Satisfied*
The ease of the admission (registration) process (n = 2000)	1675 (83.8)	325 (16.3)

The speed of the admission (registration) process (n = 1988)	1645 (82.7)	343 (17.3)

The timeliness with which your care was delivered (n = 1985)	1327 (66.9)	658 (33.1)

Items were dichotomized into 2 groups of "completely satisfied (7)" and "not completely satisfied (1-6)"

**Table 3:** Service Quality Items: Multidisciplinary Patient Care Team

How satisfied are you with our team in the following areas:	*Completely Satisfied*	*Not Completely Satisfied*
Helping you understand your medical condition (n = 1958)	1316 (67.2)	642 (32.8)

Explaining your treatment options (n = 1938)	1369 (70.6)	569 (29.4)

Involving you in decision making (n = 1936)	1432 (74)	504 (26)

The amount of time spent with you (n = 1959)	1414 (72.2)	545 (27.8)

Our team calling you by your name (n = 1956)	1699 (86.9)	257 (13.1)

Our staff genuinely caring for you as an individual (n = 1963)	1666 (84.9)	297 (15.1)

CTCA providing you with a sense of well-being (n = 1950)	1550 (79.5)	400 (20.5)

Our "whole person" approach to patient care (n = 1937)	1590 (82.1)	347 (17.9)

CTCA medical oncologist (n = 1915)	1487 (77.7)	428 (22.3)

Items were dichotomized into 2 groups of "completely satisfied (7)" and "not completely satisfied (1-6)"

**Table 4:** Patient endorsement of CTCA for themselves and others (N = 1963)

Item	Categories	N	%
Will you recommend CTCA to friends and associates?	Not at all likely	3	0.1
	1	3	0.1
	2	1	0.05
	3	1	0.05
	4	2	0.1
	5	20	1.0
	6	10	0.5
	7	39	1.9
	8	98	4.9
	9	233	11.5
	Extremely Likely	1553	77.0

### Univariate Analysis - Predictors of Patient Willingness to Recommend

Kendall's tau b correlations between the service quality measures and willingness to recommend were all significant at p < .05, with values ranging from 0.20 to 0.40 (see Table 5). Univariate logistic regression analyses were also all significant at p < .05, with odds ratios ranging from 3.2 to 9.5 (see Table 6)., In addition, prior treatment history was found to be predictive of patient willingness to recommend such that newly diagnosed patients were more likely to recommend as compared to those who had been previously treated. Age and gender were not significant.

**Table 5:** Association between Patient Endorsement of CTCA and Service Quality Measures

Variable	Kendall's tau b	P-value
The ease of the admission (registration) process	0.20	**< 0.001**

The speed of the admission (registration) process	0.22	**< 0.001**

The timeliness with which your care was delivered	0.29	**< 0.001**

Helping you understand your medical condition	0.40	**< 0.001**

Explaining your treatment options	0.40	**< 0.001**

Involving you in decision making	0.38	**< 0.001**

The amount of time spent with you	0.38	**< 0.001**

Our team calling you by your name	0.30	**< 0.001**

Our staff genuinely caring for you as an individual	0.38	**< 0.001**

CTCA providing you with a sense of well-being	0.44	**< 0.001**

Our "whole person" approach to patient care	0.38	**< 0.001**

CTCA medical oncologist	0.37	**< 0.001**

Gender (female as referent group)	0.04	0.10

Treatment History (previously treated as referent group)	-0.09	**< 0.001**

Age (used as continuous variable)	0.03	0.17

**Table 6:** Univariate Logistic Regression Analysis

Variable	OR	95% CI	P-value
The ease of the admission (registration) process	3.2	2.4 to 4.1	**< 0.001**

The speed of the admission (registration) process	3.5	2.7 to 4.5	**< 0.001**

The timeliness with which your care was delivered	4.1	3.3 to 5.2	**< 0.001**

Helping you understand your medical condition	7.5	5.9 to 9.6	**< 0.001**

Explaining your treatment options	7.2	5.7 to 9.2	**< 0.001**

Involving you in decision making	6.5	5.1 to 8.3	**< 0.001**

The amount of time spent with you	6.5	5.1 to 8.3	**< 0.001**

Our team calling you by your name	5.6	4.2 to 7.4	**< 0.001**

Our staff genuinely caring for you as an individual	7.9	6.1 to 10.4	**< 0.001**

CTCA providing you with a sense of well-being	9.5	7.4 to 12.2	**< 0.001**

Our "whole person" approach to patient care	7.3	5.6 to 9.4	**< 0.001**

CTCA medical oncologist	6.6	5.2 to 8.5	**< 0.001**

Gender (female as referent group)	0.83	0.67 to 1.03	0.10

Treatment History (previously treated as referent group)	1.6	1.3 to 2.0	**0.001**

Age (used as continuous variable)	1.006	0.99 to 1.02	0.27

### Multivariate Analysis - Predictors of Patient Willingness to Recommend

Before proceeding with multivariate analysis, we checked the bivariate Kendall's tau b correlation among the service quality predictors to screen for observable multicollinearity. Speed of admission and ease of admission were highly correlated (tau b = 0.74). "Explaining treatment options" was highly correlated with several items ("helping you understand your condition", tau b = 0.77 and "involving you in decision-making", tau b = 0.74). "Providing a sense of well being" and "caring for you as an individual" were highly correlated (tau b = 0.70). "Ease of admission", "explaining treatment options", and "providing a sense of well being" were accordingly not used in the multivariate model. "Ease of admission" and "providing a sense of well being" were dropped because we believe they may not have been consistently interpreted by patients. "Explaining treatment options" was dropped because it was highly correlated with several items and so dropping it was the most parsimonious approach.

Table 7 displays the results of the multivariate logistic regression. The overall model was significant (chi-square 426.0, df = 16, p < .001). The service quality items that were significant in the final model were "team helping you understand your medical condition", "staff genuinely caring for you as an individual" "whole person approach to patient care" and "CTCA medical oncologist." Odds ratios ranged from about 2.0 to 2.2 for these service quality measures. Gender, treatment history, CTCA center and survey year were also significant. Males had lower willingness to recommend than females. Newly diagnosed patients had higher willingness to recommend as compared to those who were previously treated. Patients surveyed in 2009 were more like to recommend as compared to those surveyed in 2007. Patients surveyed at CTCA Southwestern in Tulsa, OK, were more likely to recommend as compared to those treated at CTCA Midwestern in Zion, IL, and CTCA Eastern at Philadelphia, PA. Finally, the type of cancer diagnosis was not found to influence patient "willingness to recommend" in the multivariate model. VIF values for the service quality measures ranged from 1.3 to 2.5, none of which indicate a significant problem with multicollinearity [24,25].

**Table 7:** Multivariate Logistic Regression Analysis

Variable	OR	95% CI	P-value
The speed of the admission (registration) process	1.3	0.91 to 1.9	0.15

The timeliness with which your care was delivered	1.4	0.98 to 1.9	0.06

Helping you understand your medical condition	2.2	1.5 to 3.2	**< 0.001**

Involving you in decision making	1.2	0.82 to 1.8	0.31

The amount of time spent with you	1.3	0.87 to 1.9	0.20

Our team calling you by your name	0.82	0.52 to 1.3	0.38

Our staff genuinely caring for you as an individual	2.0	1.3 to 3.0	**0.001**

Our "whole person" approach to patient care	2.0	1.4 to 2.9	**< 0.001**

CTCA medical oncologist	2.2	1.6 to 3.1	**< 0.001**

Gender (female as referent group)	0.68	0.51 to 0.91	**0.009**

Treatment History (previously treated as referent group)	1.5	1.1 to 1.9	**0.01**

Age (used as continuous variable)	0.99	0.98 to 1.01	0.81

CTCA Center (overall effect)			0.02
Midwestern versus Southwestern	0.71	0.51 to 0.99	**0.04**
Eastern versus Southwestern	0.60	0.41 to 0.88	**0.009**

Survey Year (overall effect)			0.03
2008 versus 2007	1.00	0.72 to 1.4	0.98
2009 versus 2007	1.50	1.1 to 2.2	**0.02**

## Discussion

Patient-reported service quality assesses the extent to which an individual's health care experiences match his or her expectations which in turn can influence a patient's willingness to recommend a health care provider to friends and associates. The present study investigates this association in an acute care national oncology hospital network.

Our findings show that helping a patient to understand her/his condition, caring for a patient as an individual, a whole-person approach to care, and satisfaction with the medical oncologist all contribute to willingness to recommend CTCA to friends and associates. On the other hand, speed of admission, timeliness with which care was delivered, involving a patient in decision-making, calling a patient by their name, and the amount of time spent with a patient may not be as critical in willingness to recommend, relative to the other measures studied. These findings suggest that service quality that is central to the patient experience is critical for patient loyalty. The only seeming exceptions to this are amount of time spent with a patient and involving a patient in decision-making, but in this population, patients find these of lesser import than the quality of care itself. Further studies would need to examine these factors in new patient populations.

In order to put our study in context, we review here a few available studies in the healthcare literature which have investigated service quality predictors of patient willingness to recommend a healthcare provider. A study conducted in 1910 patients in clinics throughout Taiwan investigated whether attributes of perceived clinic quality and patient education were associated with patient satisfaction and recommendation of a primary care provider. Patient recommendation was measured on a five-point Likert scale using the question 'When your family, relatives or friends need to see a doctor, would you recommend this clinic?' The study found doctor's technical skill to be the most critical attribute of primary care quality for both overall satisfaction and recommendation, followed by doctor's interpersonal skill [9]. Another study conducted in 4945 patients in 126 Taiwanese hospitals examined the correlation of patient satisfaction with and recommendation of a hospital to patient ratings of interpersonal and technical performance of the hospital. Patient recommendation was measured on a five-point Likert scale using the question 'If someone asks you about the hospital, would you recommend it?' The study found that technical competence was a more influential predictor for recommendation [10]. Another study conducted in 2160 consecutive adult patients treated within 36 family practice clinics in Slovenia investigated factors influencing patients' recommendation of doctor. Patients' responded to the statement "I can strongly recommend my family doctor to my friends" on a five-point scale, from strongly disagree to strongly agree. Higher satisfaction with doctor's working style and organization of the health care system predicted patient recommendation [26].

The results of our study do not compare directly with above mentioned studies because of the differences in study design, patient population studied, questionnaire used and factors adjusted for. Nevertheless, our study adds useful information to the growing body of literature on the importance of assessing patient perception about service quality as a predictor of patient willingness to recommend a hospital.

Although this study reports on a relatively uncommon analysis of predicting patient willingness to recommend with perceived service quality, several limitations of the study require acknowledgment. The patient cohort was limited to only those patients who were English speakers, so this study sample is therefore not broadly representative of cancer patients in general. As a result, the generalizability of this study is limited. The data we used for this study were not primarily meant for research purposes. CTCA is a unique medical center. It specializes in treating only cancer patients, and it has an intense focus on patient-centered care. Compared to other centers, patients report very high levels of service quality at CTCA. Our study, which is hypothesis generating by nature, used a non-validated patient satisfaction questionnaire. However, it is reasonable to use a non-validated survey if the intent of the study is hypothesis generation rather than hypothesis testing. It might be argued that patients do not have the ability to judge a hospital's performance; however, patient perception is a key factor for hospital selection. This was the main goal of our study - to show the effects of patient perception about service quality on patient recommendation of a hospital. Finally, a response rate of 73.3% could potentially introduce a selection bias in our study. The baseline characteristics of patients who did not respond are not available for us to evaluate any systematic differences between responders and non-responders.

More and more health care consumers are using the web to research and shop for the best health care providers, especially for complex medical conditions. In addition, in our own experience, we hear about more and more patients traveling great distances to receive care, due in large part, to a strong recommendation of the provider from the patient's friend, associate, colleague, or family member. And as the asymmetry in information between providers and consumers decreases, we can and should expect to see more informed consumers shopping for the best available healthcare.

The strengths of our study include: a large sample size, the fact that we measured service quality as close to the time service was delivered as possible, and the fact that we used willingness to recommend (using the question and scale most commonly used in industry) as our dependent variable, which has been previously validated through research in many industries. To the best of our knowledge, this study is the first in the health care literature to report on the positive correlation between patient-reported service quality and patient willingness to recommend a provider in a large sample of cancer patients.

During a time in which quality and value are becoming increasingly prominent themes in healthcare reform, we believe the patient's perspective of the key drivers of loyalty should be given greater national attention. In most of American medicine, we assess the relative importance of quality and patient satisfaction measures from expert panels and other traditional research methods developing institutionalized views of the attributes of health care that are most important. But what has been largely missing in the conversation is the patient's perspective (and perception) of the relative importance of key aspects of service quality in the health care delivery cycle. These are areas that require further research and at the time of this writing, our organization is seeking partners to conduct national population-based research on the key drivers of value in oncology, with service quality being an important dimension to be studied. As the health care legislation continues to be implemented, the entire health care system would benefit from a greater focus on the key drivers of value from the consumer's perspective - the patient, as well as their caregivers and families. These are important areas of research that will lend greater focus to where, when, and how we should apply our scarce resources to deliver the most valuable care to our patients - the ultimate consumer.

Next steps in our research include linking data on service quality to patient outcomes. We are unaware of any literature linking service quality to data on patient quality of life, length of life, and overall satisfaction with health. Research is also underway at our center to explore the relationship between patient willingness to recommend and actual patient return (behavior) as well as how changes in patients' clinical condition affect their willingness to recommend a provider, controlling for all other known variables. With respect to population-based research, we do plan on conducting national research on the patient's perspective of value in oncologic care, data that has largely been missing from the health policy discussions.

## Conclusions

In this multi-center study, we demonstrate the predictive significance of perceived service quality as it relates to patient willingness to recommend an oncology service provider. We identified four key service quality drivers of patient loyalty: "team helping you understand your medical condition", "staff genuinely caring for you as an individual" "whole person approach to patient care" and "CTCA medical oncologist".

## Competing interests

The authors declare that they have no competing interests.

## Authors' contributions

CGL participated in concept, design, writing, statistical analysis and data interpretation. MR participated in concept, statistical analysis and data interpretation. DG participated in concept, statistical analysis, data interpretation and writing. All authors read and approved the final manuscript.

## Pre-publication history

The pre-publication history for this paper can be accessed here:

http://www.biomedcentral.com/1472-6963/11/46/prepub
